# Inhibition of IGFBP4 in Granulosa Cells Improves Reproductive Performance and Maintains Fertility With Age via YAP Signaling

**DOI:** 10.1002/advs.202510226

**Published:** 2026-07-16

**Authors:** Qianhui Hu, Ajun Geng, Ziyuan Li, Fanghao Guo, Jingqiang Wang, Xinyi Chen, Meiling Zhang, Yi Arial Zeng, Wen Li

**Affiliations:** ^1^ Center For Reproductive Medicine & Fertility Preservation Program School of Medicine International Peace Maternity and Child Health Hospital Shanghai Jiao Tong University Shanghai China; ^2^ Shanghai Key Laboratory of Embryo Original Disease Shanghai China; ^3^ State Key Laboratory of Cell Biology CAS Center for Excellence in Molecular Cell Science Institute of Biochemistry and Cell Biology Chinese Academy of Sciences University of Chinese Academy of Sciences Shanghai China; ^4^ The Center of Reproductive Medicine Shanghai Changzheng Hospital Naval Medical University Shanghai China; ^5^ Hospital of Zhejiang People's Armed Police Hangzhou China; ^6^ Key Laboratory of Systems Health Science of Zhejiang Province, School of Life Science, Hangzhou Institute for Advanced Study, Hangzhou University of Chinese Academy of Sciences China

**Keywords:** female fertility, granulosa cells, IGFBP4, ovarian aging, yes‐associated protein signaling

## Abstract

Ovarian aging is a critical factor influencing reproductive capacity and overall health. Granulosa cells (GCs) play essential roles in folliculogenesis; however, the mechanisms by which GC dysfunction contributes to ovarian aging remain incompletely understood. In this study, we identified insulin‐like growth factor binding protein 4 (IGFBP4) as a negative regulator of ovarian function that is upregulated in GCs from aged cynomolgus monkey ovaries. Using an *Igfbp4*‐HA tagged mouse model, we found that IGFBP4 expression in GCs increased during follicle development and was further elevated in aged mice. RNA‐seq analysis of *Igfbp4*‐deficient GCs revealed activation of the YAP pathway, which supports follicular development. Mechanistically, IGFBP4 reduced YAP nuclear localization in GCs, thereby restraining downstream YAP target gene expression and GC proliferation. In *Amhr2‐Cre; Igfbp4^fl/fl^* mice, GC‐specific deletion of *Igfbp4* enhanced folliculogenesis, increased litter size and preserved reproductive performance with age. Elevated IGFBP4 levels were also detected in GCs from aging women and patients with premature ovarian insufficiency (POI). Furthermore, higher concentrations of IGFBP4 were observed in the follicular fluid of POI patients, supporting its potential as a biomarker of ovarian dysfunction. These findings establish IGFBP4 as a GC‐derived suppressor of ovarian function and a potential target for preserving ovarian function during aging.

## Graphical abstract

IGFBP4 is upregulated in granulosa cells of aged ovaries across monkeys, mice, and humans. It inhibits YAP signaling, thereby suppressing cell proliferation and contributing to follicular dysfunction. Deletion of *Igfbp4* in granulosa cells enhances ovulatory output, improves hormone profiles, and reproductive performance in aged female mice, suggesting IGFBP4 as a therapeutic target for improving ovarian function during aging.

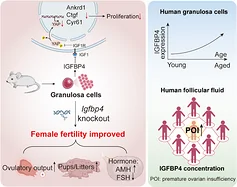

## 1 Introduction

The ovary is among the earliest organs to undergo functional aging in mammals, resulting in a decline in female reproductive capacity and ultimately menopause. As a key regulator of female reproductive and endocrine function, ovarian deterioration is associated with accelerated systemic aging and increase the risk of multiple age‐related disorders, including cardiovascular disease, diabetes, bone loss, and fractures [1], thereby affecting both women's reproductive health and overall well‐being. Despite medical advances that have extended human lifespan, the duration of ovarian function has remained largely unchanged [2]. Meanwhile, the demand for interventions targeting age‐related female infertility has gradually increased. However, the molecular mechanisms underlying ovarian aging remain incompletely understood.

The ovarian follicle, composed of an oocyte surrounded by granulosa cells (GCs), is the basic functional unit of female reproduction. Ovarian reserve refers to the quantity and developmental potential of the remaining follicles in the ovary, which constitute the non‐renewable pool of oocytes established before birth. This reserve gradually diminishes with age owing to the continuous loss of follicles through atresia and ovulation, ultimately leading to a decline in female fertility. GCs play a pivotal role in follicular growth and oocyte maturation by producing essential hormones and paracrine factors, including anti‐Müllerian hormone (AMH), KIT ligand (KITL), C‐type natriuretic peptide (CNP), insulin‐like growth factor 1 (IGF1), vascular endothelial growth factor (VEGF), bone morphogenetic protein‐6 (BMP6), pituitary adenylate cyclase‐activating polypeptide (PACAP), and vasoactive intestinal polypeptide (VIP) [3, 4, 5, 6]. As oocytes grow, GCs undergo rapid proliferation and subsequently differentiate into cumulus cells (CCs) and mural granulosa cells (MGCs) [6] to support meiotic progression and follicular expansion. However, excessive or dysregulated apoptosis of GCs can trigger follicular atresia, thereby contributing to the depletion of ovarian reserve [4, 7]. Given their central role in folliculogenesis and ovarian homeostasis, we focused on granulosa cells to define age‐associated transcriptional changes and mechanisms relevant to ovarian aging.

The IGF signaling pathway is crucial for development, metabolism, and cell proliferation. IGF1 activate downstream signaling by binding to insulin‐like growth factor receptors (IGF1R) through receptor autophosphorylation. The activity of IGFs is regulated by insulin‐like growth factor binding proteins (IGFBPs) [8, 9], which can either inhibit or potentiate IGF activity. For example, IGFBP1 can stimulate dermal wound healing by activating IGF1 [10], whereas IGFBP4 inhibits IGF‐induced regulatory T‐lymphocyte induction in human mesenchymal stem cells [11]. In addition, IGFBPs are reported to exert IGF‐independent actions [12], as they can bind to cell surface receptors and activate intracellular signaling pathways. For instance, IGFBP4 promotes cardiomyocyte differentiation from stem cells by suppressing β‐catenin signaling [13] and acts as a cardiogenic growth factor by inhibiting Wnt signaling [14].

The IGF signaling pathway also plays a pivotal role in the development of ovarian follicles. GCs express IGF1 and IGF1R, both of which contribute to follicular development and steroidogenesis [15]. IGF1R is essential for GC survival, and its loss leads to increased apoptosis [16]. Interestingly, studies in ruminants have reported that small IGFBPs (<40 kDa), including IGFBP2, IGFBP4, and IGFBP5, are diminished in preovulatory follicles but increased in atretic follicles [17], suggesting a potential role of IGFBPs in follicular atresia. While these patterns implicate IGFBPs in atresia, the specific role of IGFBP4 in GC function and its involvement in ovarian aging remain insufficiently understood. Notably, mechanistic studies on IGFBP4 are scarce in both murine and primate models, highlighting the need for cross‐species investigations to elucidate its functional significance in folliculogenesis and reproductive decline.

Yes‐associated protein (YAP), a key effector of the Hippo pathway, is an important regulator of granulosa cell proliferation and ovarian follicle development [18]. In this study, we compared single‐cell RNA sequencing (scRNA‐seq) data from juvenile and aged cynomolgus monkey ovaries and identified a marked upregulation of IGFBP4 in aging GCs. We further explored its functional role and demonstrated that IGFBP4 suppresses GC proliferation by attenuating YAP signaling. Conditional deletion of *Igfbp4* in GCs enhanced follicle development and improved reproductive performance in mice. Furthermore, IGFBP4 expression was elevated in GCs from aged women and patients with premature ovarian insufficiency (POI), underscoring its clinical relevance and identifying IGFBP4 as a potential target for preserving ovarian function during aging.

## 2 Results

### 2.1 IGFBP4 is Highly Expressed in Aged Ovaries and During Follicular Atresia in Cynomolgus Monkeys and Mice

To investigate how GCs contribute to ovarian aging, we first re‐analyzed publicly available scRNA‐seq data from cynomolgus monkey ovaries spanning juvenile (4–5 years) and aged (18–20 years) stages (Figure 1A,B and Figure S1A) [19]. The GCs cluster was identified based on AMHR2 and AMH expression and subsequently separated into young and aged groups (Figure 1C and Figure S1B). Although young and aged GCs showed only modest separation on the UMAP, differential expression analysis identified clear age‐associated molecular changes, consistent with the gradual and continuous nature of ovarian aging (Figure 1D). Given the critical role of IGF signaling in follicular development, we focused on genes related to the IGF family and the pathway components. Among them, *IGFBP4* was significantly upregulated in aged granulosa cells compared with their younger counterparts (Figure 1D and Figure S1C).

**FIGURE 1:**
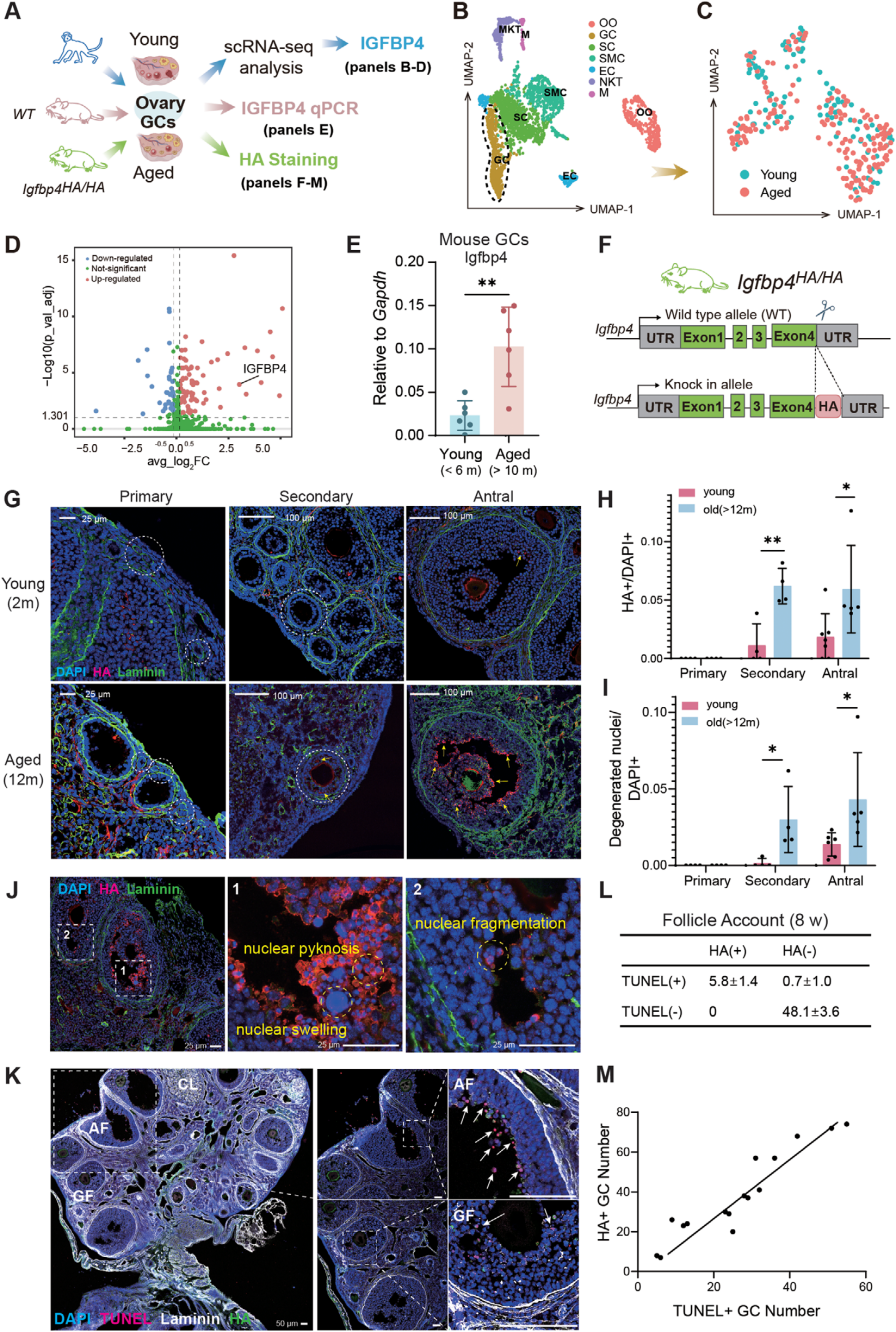
IGFBP4 is highly expressed in aged ovaries and atretic follicles. (A) Illustration of the experimental design. The ovarian granulosa cells (GCs) of young and aged non‐human primates (NHPs) were analyzed by single‐cell RNA sequencing (scRNA‐seq). Additionally, GCs from young and aged wild‐type mice and *Igfbp4^HA/HA^* mice were analyzed by qPCR and hemagglutinin (HA) staining, respectively. (B, C, and D) Transcriptional signatures determined by single‐cell RNA sequencing (scRNA‐seq) analysis of ovarian tissues from both young (4–5 years old) and aged (18–20 years old) cynomolgus monkeys. Uniform manifold approximation and projection (UMAP) plot showing seven major ovarian cell types: oocyte (OO), granulosa cell (GC), stromal cell (SC), smooth muscle cell (SMC), endothelial cell (EC), natural killer T cell (NKT), and macrophage (M) (B). GCs were further divided into young and aged primate cell populations (C). Volcano plot showing differentially expressed genes (DEGs) between aged and young granulosa cells. Upregulated expression of IGFBP4 in aged cells is highlighted. The horizontal dotted line represents the adjusted p‐value threshold (*p*  =  0.05), and the vertical dotted lines represent log_2_ fold change cutoffs (± 0.5) (D). (E) qPCR analysis of mouse GCs showed the expression of *Igfbp4* in young (2‐month) and aged groups (12‐month) (*n* = 6). Mean ± SD, ^**^ *p* < 0.01. (F) Schematic diagram showed the strategy to generate an *Igfbp4^HA/HA^* mouse model. (G) Representative immunofluorescence images showing IGFBP4‐HA expression (red), laminin (green), and DAPI (blue) in primary, secondary, and antral follicles from young (2‐month‐old) and aged (12‐month‐old) mice. (H) Quantification of IGFBP4‐HA‐positive granulosa cells (HA^+^/DAPI^+^) across stages. Mean ± SD. ^*^ *p* < 0.05, ^**^ *p* < 0.01. (I) Proportion of granulosa cells with degenerated nuclei (pyknotic/fragmented/swollen nuclei) among total DAPI^+^ cells. Mean ± SD. ^*^ *p* < 0.05. (J) *Igfbp4* was enriched within granulosa cells exhibiting nuclear pyknosis, fragmentation, or swelling (yellow dotted circles). Scale bar, 25 µm. (K) Immunofluorescence staining of the HA label and terminal deoxynucleotidyl transferase dUTP nick end labeling (TUNEL) staining confirming enrichment of Igfbp4 expression in atretic follicles (n = 5). Scale bar, 50 µm. (L) Summary table showing the overlap between *Igfbp4‐HA*‐positive follicles and TUNEL‐positive atretic follicles in 8‐week‐old mice (*n* = 5). (M) The number of *Igfbp4* positive GCs (HA+ GC number) is positively correlated with the number of apoptotic GCs (TUNEL+ GC number).

To further validate the age‐associated increase of *Igfbp4* observed in primate transcriptomic data, we examined its expression in a well‐established mouse model. GCs were isolated from young (<6 months) and aged (>10 months) mice using a previously established FACS strategy that enriches granulosa cells by gating the mTomato low fluorescence (mTom‐low) population from *Rosa26^mTmG^* ovaries [20]. In *Rosa26^mTmG^* mice, mTomato (mTom) fluorescence is dimmer within follicles than in the surrounding ovarian tissue (Figure S1D), enabling GC enrichment using the mTom‐low gate. Confocal imaging, in‐culture morphology, and quantitative polymerase chain reaction (qPCR) analyses of GC marker genes (*Cyp19a1*, *Foxl2*, *Esr1*, *Esr2*, and *Fshr*) collectively confirmed that the mTom‐low population corresponded to granulosa cells (Figure S1E,F). Using these purified GCs, we performed qPCR and found that *Igfbp4* expression was significantly higher in GCs from aged mice than in those from young mice (Figure 1E), consistent with the transcriptomic findings in cynomolgus monkeys.

To visualize *Igfbp4* expression in vivo, we constructed an *Igfbp4*‐hemagglutinin (HA) knock‐in mouse by inserting an HA tag before the termination codon using CRISPR/Cas9 editing (Figure 1F). HA immunostaining revealed clear *Igfbp4* signals in secondary and antral follicles of young controls, while primary follicles showed weak or undetectable staining (Figure 1G). Importantly, IGFBP4‐HA expression is significantly enhanced in secondary and antral follicles in aged mice compared to the young controls (Figure 1G,H). Furthermore, *Igfbp4*‐positive GCs in aged ovaries frequently exhibited nuclear swelling, pyknosis, and fragmentation (Figure 1J), morphological features indicative of apoptotic or degenerative changes. This observation also parallels previous reports describing IGFBP4 expression in atretic follicles [21, 22]. Quantification further showed that the proportion of GCs with degenerated nuclei (pyknotic/fragmented/swollen) among total DAPI^+^ cells was significantly higher in aged mice across secondary and antral follicles (Figure 1I), confirming the enrichment of nuclear abnormalities in aged *Igfbp4‐*positive follicles. Following established morphological criteria [23, 24], primary and secondary follicles were classified as atretic when they contained one or more apoptotic GCs, whereas later‐stage follicles were classified as atretic when apoptotic features were present in more than 10% of GCs. Co‐immunostaining for HA and TUNEL in *Igfbp4‐HA* ovaries demonstrated that all HA‐positive follicles were TUNEL‐positive (Figure 1K,L). Quantitative analysis further showed a strong correlation between the number of *Igfbp4*‐expressing GCs and apoptotic GCs within individual follicles (Figure 1M). Collectively, these findings indicate that *Igfbp4* expression is most evident in GCs of growing follicles, particular in secondary and antral stages, and becomes more pronounced in aged ovaries.

### 2.2 IGFBP4 Inhibits the Proliferation of Granulosa Cells

To explore the functional role of IGFBP4, we performed both gain‐ and loss‐of‐function experiments in KGN cells (a human ovarian GC‐like tumor cell line). The addition of IGFBP4 to the KGN culture medium significantly inhibited cell proliferation, as evidenced by reduced cell numbers during passaging and decreased EdU staining. The results showed that KGN cell growth was significantly inhibited in the presence of IGFBP4 protein in a dose dependent manner, as shown by lower cell counts (Figure 2A,B) and reduced percentages of EdU^+^ cells (Figure 2A,C and Figure S1H).

**FIGURE 2:**
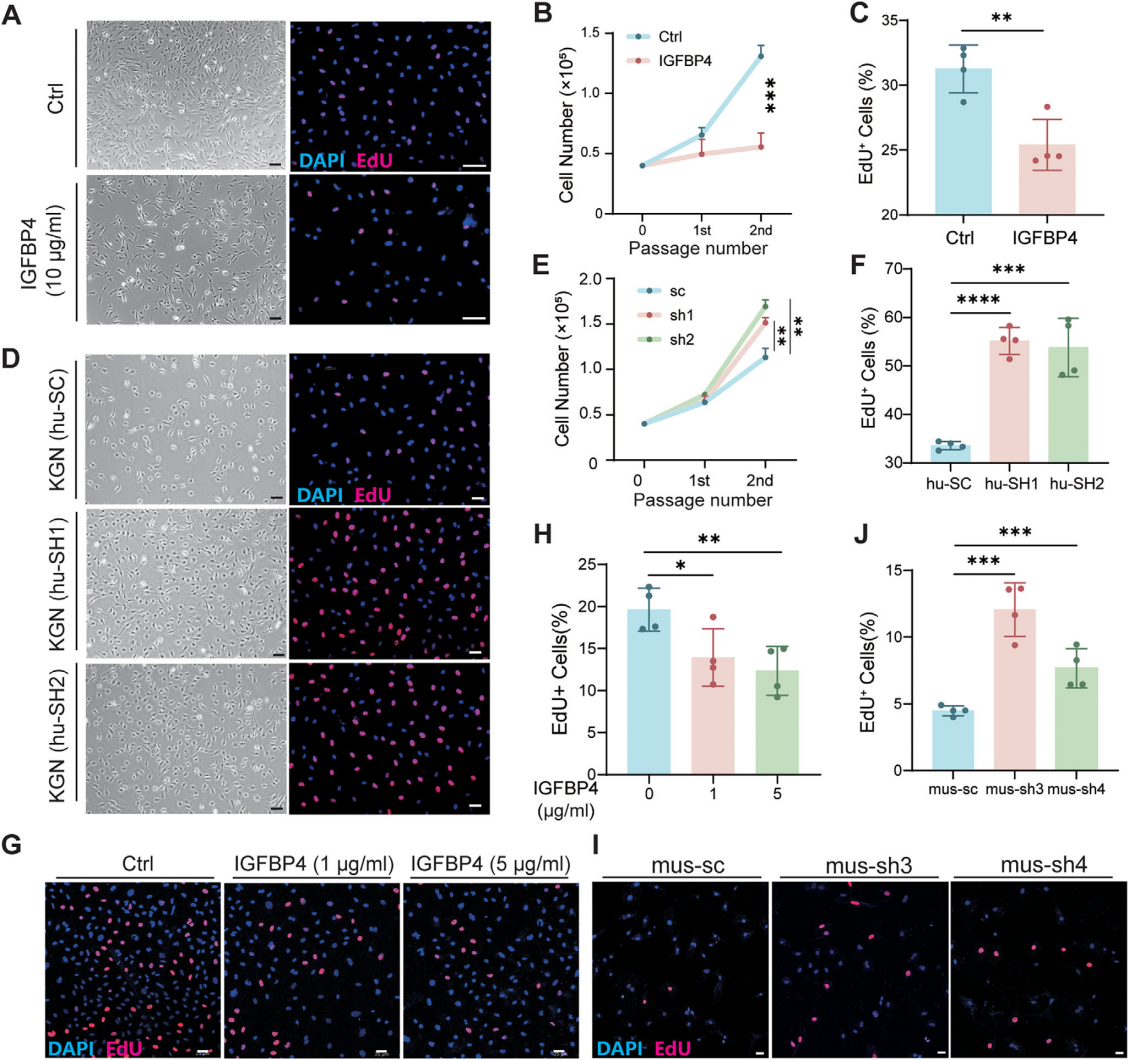
IGFBP4 inhibits the proliferation of granulosa cells. (A) Representative EdU staining images of KGN cells cultured with or without 10 µg/mL IGFBP4 protein. Scale bar, 25 µm. (B,C) The passage number and the percentage of EdU^+^ cells of KGN cell cultured with or without IGFBP4 protein (*n* = 3). Mean ± SD. ^***^ *p* < 0.001, ^**^ *p* < 0.01. (D) Representative EdU staining images of KGN cells treated with control short hairpin RNA (shRNA) (SC), shRNA1 (SH1), and shRNA2 (SH2) (*n* = 4). Scale bar, 25 µm. (E,F) The passage number and the percentage of EdU^+^ cells of the KGN cell treated with SC, SH1, SH2 (*n* = 4). Mean ± SD. ^**^ *p* < 0.01. (G, H) EdU staining and the quantification of EdU^+^ cells from primary mouse GCs treated with 0, 1 µg/mL, or 5 µg/mL IGFBP4. Data represent four independent biological replicates (*n*  =  4). Scale bar, 25 µm. Mean ± SD. ^*^ *p* < 0.05, ^**^ *p* < 0.01. (I,J) EdU staining and the quantification of EdU^+^ cells from primary mouse GCs treated with sc, shRNA3 (sh3), shRNA4 (sh4). Data represent four independent biological replicates (*n*  =  4). Scale bar, 25 µm. Mean ± SD. ^***^ *p* < 0.001.

We further investigated IGFBP4 function using short hairpin RNA (shRNA)‐mediated knockdown. Two shRNAs (SH1 and SH2) were independently used, and their knockdown efficiencies in KGN cells were validated (Figure S2A). Knockdown of *IGFBP4* promoted cell proliferation, as indicated by increased cell numbers (Figure 2D,E) and elevated percentages of EdU^+^ cells (Figure 2D,F).

We further validated this effect in mouse primary GCs. Addition of IGFBP4 protein inhibited GC growth (Figure 2G,H), whereas *Igfbp4* knockdown promoted GC proliferation (Figure 2I,J). These findings suggest that IGFBP4 negatively regulates GC proliferation.

### 2.3 IGFBP4 Inhibits the YAP Signaling Pathway in Granulosa Cells

To investigate the downstream mechanisms of *Igfbp4*, we performed RNA‐seq analysis of on ovarian GCs transduced with either control shRNA (CTRL) or *Igfbp4*‐shRNA (knockdown of *Igfbp4*; *Igfbp4*‐KD) (Figure 3A). Overall, 1,508 upregulated and 1,028 downregulated genes were identified upon *Igfbp4* knockdown (Figure S2B). Kyoto Encyclopedia of Genes and Genomes (KEGG) pathway enrichment analysis revealed that the Hippo signaling pathway was among the top 3 enriched (Figure 3B).

**FIGURE 3:**
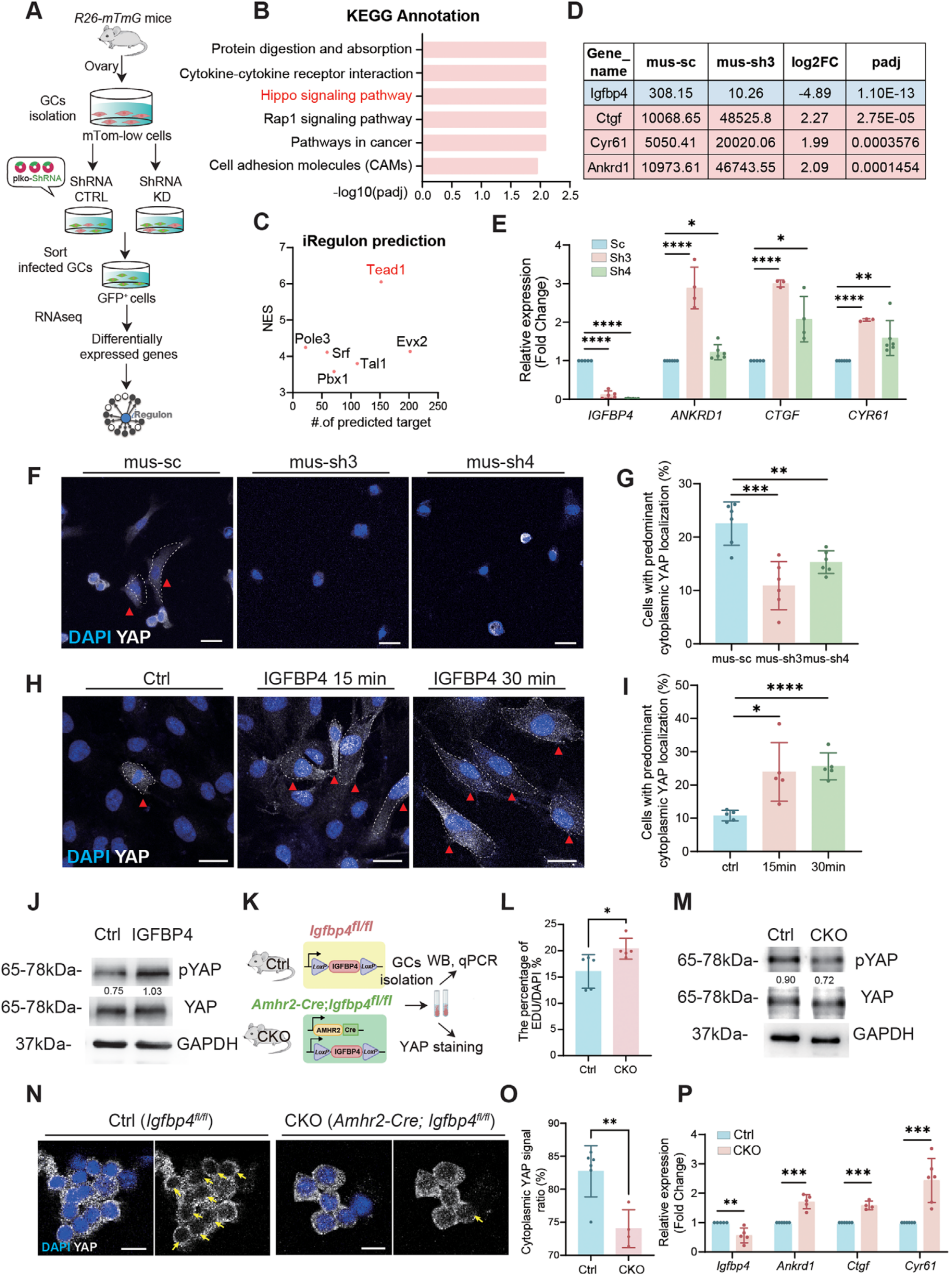
IGFBP4 inhibits YAP signaling in GCs. (A) Schematic diagram illustrating the identification of IGFBP4 downstream pathway. (B) Kyoto Encyclopedia of Genes and Genomes (KEGG) pathway enrichment analysis of *Igfbp4* knockdown GCs (GCs). (C) Transcription factors predicted from RNA‐seq data of Igfbp4‐knockdown mouse primary GCs using iRegulon. Normalized Enrichment Scores (NES). (D) Differentially expressed target genes of the yes‐associated protein (YAP) signaling pathway identified from RNA‐seq data. (E) Quantitative polymerase chain reaction (qPCR) analysis validated *Igfbp4* knockdown efficiency and the upregulation of YAP target gene expression (*n* = 6). Mean ± SD. ^*^ *p* < 0.05, ^**^ *p* < 0.01, ^***^ *p* < 0.001, ^****^ *p* < 0.0001. (F,G) Immunofluorescence staining of total YAP (F) and quantification of the percentage of cells with predominant cytoplasmic YAP localization (G) in mouse primary GCs treated with control shRNA (sc), short hairpin RNA 3 and 4 (sh3 and sh4) (*n* = 6). For each biological replicate, 3–5 random microscopic fields were analyzed. Red arrows indicate cells with predominant cytoplasmic YAP localization. Scale bar, 25 µm. Mean ± SD. ^**^ *p* < 0.01, ^***^ *p* < 0.001. (H,I) Immunofluorescence staining of total YAP (H) and quantification of the percentage of cells with predominant cytoplasmic YAP localization (I) in KGN cells treated with 5 µg/ml IGFBP4 for short‐term stimulation (15 min and 30 min) (*n* = 5). For each biological replicate, 3–5 random microscopic fields were analyzed. Red arrows indicate cells with predominant cytoplasmic YAP localization. Scale bar, 25 µm. Mean ± SD. ^*^ *p* < 0.05, ^****^ *p* < 0.0001. (J) Western blot analysis showing the effect of IGFBP4 stimulation on YAP phosphorylation in KGN cells. KGN cells were harvested after 30 min of stimulation with 5 µg/mL IGFBP4 protein. The numbers shown below the bands indicate the relative P‐YAP/YAP values of the representative blot. (K) A schematic representation of *Igfbp4^fl/fl^* mice (Ctrl) and *Amhr2‐Cre*;*Igfbp4^fl/fl^* (CKO) mice. All experimental mice were homozygotes for the floxed *Igfbp4* allele. (L) Quantification of EdU^+^ granulosa cells isolated from ovaries of control and IGFBP4 CKO mice (*n* = 3). Mean ± SEM. ^*^ *p* < 0.05. (M) Western blot analysis of YAP phosphorylation in primary ovarian GCs isolated from Ctrl and CKO mice. Numbers below the bands indicate relative P‐YAP/YAP ratios in the representative blot. (N, O) Immunofluorescence staining showing total YAP localization in primary ovarian GCs isolated from Ctrl and CKO mice (N). Yellow arrows indicate cells with high cytoplasmic YAP signal. The cytoplasmic YAP signal ratio was quantified as the cytoplasmic YAP integrated density divided by the total cellular YAP integrated density (O). Scale bar, 10 µm. Mean ± SD. ^**^ *p* < 0.01. (P) qPCR analysis validated *Igfbp4* knockdown efficiency in GCs from CKO mice and the upregulation of YAP target gene in vivo (*n* = 5). Mean ± SD. ^*^ *p* < 0.05, ^**^ *p* < 0.01.

To further dissect regulatory factors, we applied iRegulon, which identified TEA‐domain transcription factor 1 (TEAD1), the major transcription factor of the Hippo/YAP pathway, as the top‐scoring regulator based on the normalized enrichment score (NES) (Figure 3C). Given that YAP/TAZ act as transcriptional co‐activators for TEADs [25], we examined canonical YAP‐responsive genes. Several well‐established YAP targets, including *Ankrd1*, *Ctgf*, and *Cyr61*, were significantly upregulated in *Igfbp4*‐KD GCs (Figure 3D) [26, 27]. qPCR validation using two non‐overlapping shRNAs confirmed the same induction pattern (Figure 3E).

YAP transcriptional activity is tightly regulated by its subcellular localization [28, 29]. We therefore examined the subcellular distribution of total YAP. In primary GCs transduced with *Igfbp4* shRNAs, the proportion of cells with predominant cytoplasmic YAP localization was reduced (Figure 3F,G). Conversely, short‐term stimulation with IGFBP4 protein for 15 or 30 min increased cytoplasmic YAP localization in KGN cells (Figure 3H,I). Treatment with 5 µg/mL IGFBP4 for 30 min also increased YAP phosphorylation in KGN cells (Figure 3J and Figure S2C). To test whether YAP activity contributes to the proliferative effect of IGFBP4 loss, we treated IGFBP4‐knockdown KGN cells with verteporfin. Verteporfin attenuated the increase in EdU incorporation induced by *IGFBP4* knockdown (Figure S2D,E). Together, these results link IGFBP4 loss to enhanced YAP nuclear localization and support a role for YAP activity in the resulting proliferative response.

We subsequently validated the IGFBP4‐YAP signaling axis in vivo. A conditional *Igfbp4* knockout allele was generated by deleting exon 1 upon Cre‐mediated recombination (Figure S2F). Using the GC‐specific *Amhr2* (anti‐Müllerian hormone receptor type 2)*‐Cre* line [30, 31], we generated GC‐specific *Igfbp4* knockout mice (*Amhr2‐Cre; Igfbp4^fl/fl^*) and control mice (*Igfbp4^fl/fl^*) (Figure 3K). To assess GC proliferation in vivo, each mouse received an intraperitoneal injection of EdU (2.5 µg/µL, 100 µL per 25 g body weight). Six hours later, ovarian GCs were isolated, plated onto slides, and subjected to EdU fluorescence staining. Quantification showed a higher proportion of EdU^+^ granulosa cells in CKO mice than in controls (Figure 3L). Next, we examined whether this proliferative phenotype was associated with altered YAP subcellular localization. Western blot analysis of isolated ovarian GCs showed reduced phosphorylated YAP levels in CKO mice, whereas total YAP protein levels were unchanged compared with controls (Figure 3M). Immunofluorescence staining further revealed a decreased cytoplasmic YAP signal ratio in CKO GCs, indicating enhanced nuclear localization of YAP upon *Igfbp4* deletion (Figure 3N,O). In addition, the canonical YAP target genes *Ankrd1, Ctgf*, and *Cyr61* were significantly upregulated in CKO ovaries compared with controls (Figure 3P). Together, these results suggest that *Igfbp4* acts as a suppressor of GC proliferation by reducing the nuclear localization of YAP and subsequently dampening YAP signaling activity.

### 2.4 Deletion of IGFBP4 Promotes Folliculogenesis and Enhances Reproductive Performance

To investigate the in vivo impact of *Igfbp4* on female fertility, we used *Amhr2‐Cre; Igfbp4^fl/fl^* (CKO) and *Igfbp4^fl/fl^* (Ctrl) mice (Figure 4A). To minimize variability due to the estrous cycle, ovaries were collected from all mice at proestrus stage. Follicle counts in 8‐week‐old adult mice showed that CKO mice had significantly higher numbers of primordial (p = 0.0043), primary (*p* = 0.0095), and secondary *(p* = 0.0019) follicles compared with Ctrl (Figure 4B,C). In parallel, CKO ovaries displayed fewer atretic follicles and more corpora lutea (p = 0.0369 and 0.0401), indicating improved follicle survival and enhanced ovulatory activity. These findings suggest that *Igfbp4* deletion in GCs promotes follicular development from the early stages and prevents premature follicular loss.

**FIGURE 4:**
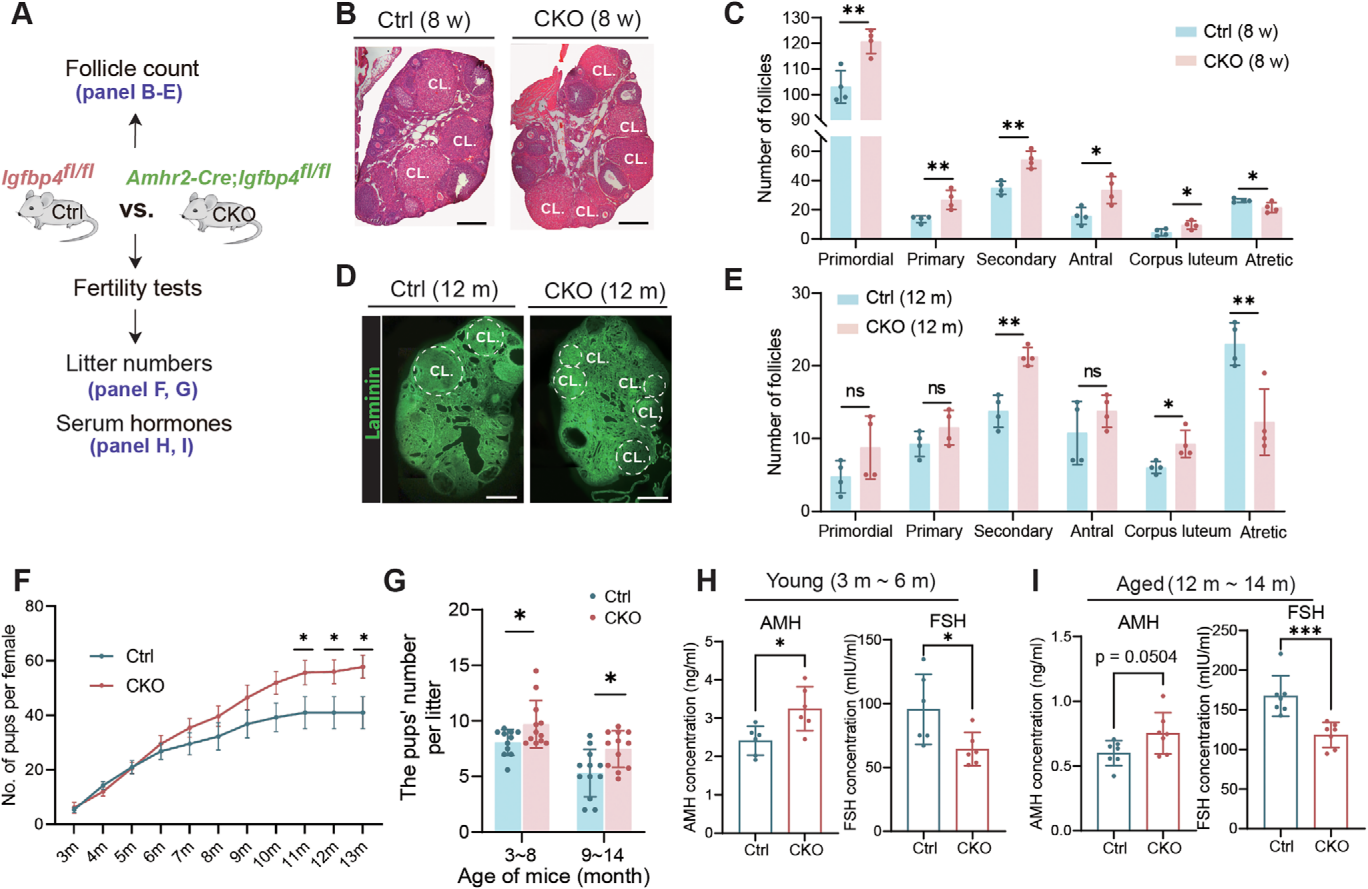
Deletion of *Igfbp4* promotes folliculogenesis and enhances reproductive performance. (A) A schematic representation of the deletion of *Igfbp4* by Amhr2Cre‐mediated recombination in the ovarian GCs of *Amhr2‐Cre; Igfbp4^fl/fl^* (CKO) mice. (B) Hematoxylin and Eosin (HE) staining of ovarian sections from 8‐week‐old control (Ctrl) and CKO mice (*n* = 3). Scale bar, 100 µm. (C, E) Quantification of ovarian follicle stages in Ctrl and *Igfbp4* CKO mice at 8 weeks (C) and 12 months (E) of age (*n* = 4 mice per group). Mean ± SD. ^*^ *p* < 0.05. ^**^ *p* < 0.01, ns, not significant. (D) Immunofluorescence staining of ovarian sections from 12‐month‐old Ctrl and CKO mice (*n* = 3). Scale bar, 100 µm. (F) Cumulative reproductive output in the continuous mating assay. Weekly pup counts were accumulated for each female and plotted as the cumulative number of pups per female at each month of age (*n* = 7 females/group). Mean ± SEM. *p < 0.05. (G) Mean litter size by age window. Each dot represents one female and is calculated as total pups divided by number of litters within the specified age window. Data for the 3–8‐month window were obtained from the original breeding cohort (*n* = 12 females per group). For the 9–14‐month window, 7 females per group from the original cohort were supplemented with 5 additional aged Ctrl and CKO females, resulting in 12 females per group. Mean ± SD. ^*^ *p* < 0.05. (H,I) Enzyme‐linked immunosorbent assay (ELISA) assay measurement of serum Anti‐Müllerian hormone (AMH) and follicle‐stimulating hormone (FSH) levels in young mice (3‐6 months) (H) and aged mice (12–14 months) (I) (young mice *n* = 6, aged mice n = 7). Mean ± SD. ^*^ *p* < 0.05, ^***^ *p* < 0.001.

To assess whether these effects extend into aging, we examined ovaries from 12‐month‐old mice, a commonly used model for studying age‐related decline in ovarian function [32, 33]. Similar to the phenotype observed in young adult mice, aged CKO ovaries exhibited fewer atretic follicles and more corpora lutea (Figure 4D). Using the same refined counting method, secondary follicle numbers remained significantly elevated in CKO mice (p = 0.0010), accompanied by fewer atretic folliclesand more corpora lutea (p = 0.019 and p = 0.0075) (Figure 4E). Although primordial and primary follicle counts were not significantly different at this age, the maintenance of more advanced follicles and corpora lutea suggests that *Igfbp4* deletion helps preserve folliculogenesis during ovarian aging.

We next evaluated long‐term reproductive performance using a continuous mating assay. Initially, 12 matched CKO–Ctrl breeding cages were established, with each cage containing one CKO female, one Ctrl female, and one wild‐type male. Breeding was initiated after the females reached 8 weeks of age. During the long‐term follow‐up, some mice died, and only 7 matched cages remained available for the cumulative reproductive analysis. Based on these 7 cages, CKO females showed higher cumulative pup output over the 13‐month observation period, with significant group differences emerging at 11–13 months (Figure 4F). To better illustrate age‐dependent fertility changes, breeding outcomes were grouped into the peak reproductive phase (3–8 months) and fertility‐decline phase (9–14 months). To maintain a consistent sample size across the two age windows, 5 additional CKO and Ctrl females were included in this analysis, resulting in 12 females per group. For each female, the average litter size within each phase was calculated as the total pup number divided by the total litter number. Using this analysis, CKO females showed a significantly larger mean litter size than Ctrl females in both age windows (Figure 4G). Notably, CKO females maintained a higher litter size than Ctrl females during the fertility‐decline phase, indicating improved reproductive performance at later ages.

Endocrine measurements further supported improved ovarian function. Serum FSH levels were significantly lower in CKO mice than in controls in both young (3–6 months) and aged (12–14 months) groups (Figure 4H–I). Serum AMH levels were significantly higher in young CKO mice and remained modestly higher in aged CKO mice (p = 0.0504), suggesting better maintenance of the functional growing follicle pool. Together, these results suggest that *Igfbp4* deletion enhances follicle survival and ovulation in young mice, helps preserve growing follicles and reproductive function with age, highlighting IGFBP4 as a potential target for counteracting age‐related ovarian dysfunction.

### 2.5 The Expression of IGFBP4 is Enhanced in Aged Women and Patients With POI

To investigate the relationship between *IGFBP4* expression and ovarian aging in humans, we first analyzed published single‐cell transcriptomic data from young and aged women [34]. We found that *IGFBP4* expression was significantly higher in the GCs from aged women (47–49 years) than in those from young women (18–28 years) (Figure 5A).

**FIGURE 5:**
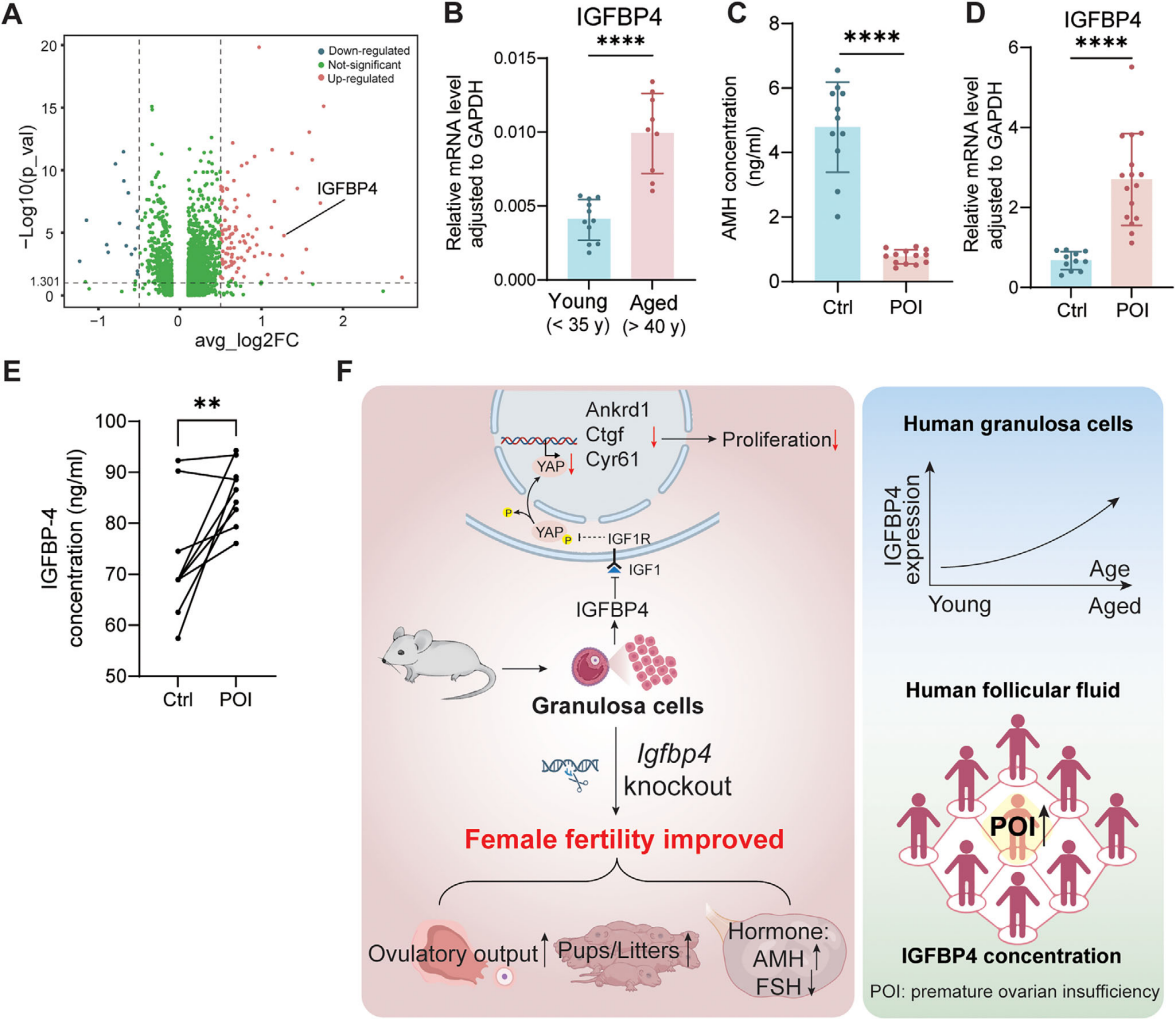
The expression of *IGFBP4* is increased in aged women and patients with premature ovarian insufficiency (POI). (A) Volcano plot showing differentially expressed genes between GCs from aged and young women based on published single‐cell RNA‐seq data. The horizontal dotted line represents the adjusted p‐value threshold (*p*  =  0.05), and the vertical dotted lines represent log_2_ fold change cutoffs (± 0.5) (B) qPCR analysis showed *IGFBP4* expression in human granulosa cells (GCs) from the aged female group (>40 years old, *n* = 9) and the young female group (≤35 years old, *n* = 12). Mean ± SD, ^****^ *p* < 0.0001. (C) Serum AMH levels were significantly reduced in POI patients (n = 13) compared with controls (*n* = 13). Mean ± SD, ^****^ *p* < 0.0001. (D) qPCR analysis showed *IGFBP4* expression in human GCs from POI patients (*n* = 11) and controls (*n* = 16). Mean ± SD, ^****^ *p* < 0.0001. (E) ELISA showed IGFBP4 concentration in follicular fluid from POI patients (*n* = 9) and age‐matched controls (*n* = 7). Mean ± SD, ^**^ *p* < 0.01. (F) An illustration depicting the mechanism and function of IGFBP4 in GCs. Created with BioRender. Hu, Q. (2025) https://BioRender.com/l4h1eqi.

We next measured IGFBP4 expression in primary GCs from women without diagnosed ovarian disease who underwent controlled ovarian stimulation and oocyte retrieval for in vitro fertilization (IVF). Patients were divided into a young (<35 years, *n* = 12) and an aged (>40 years, *n* = 9) group. IGFBP4 expression levels were significantly higher in the aged group compared to the young group (Figure 5B).

Given the strong association between GC dysfunction and premature ovarian insufficiency (POI) [35], we further assessed IGFBP4 expression in this clinical population. Serum AMH levels were markedly reduced in patients with POI compared with controls (Figure 5C). qPCR analysis showed significantly increased IGFBP4 expression in GCs from patients with POI (*n* = 16) (Figure 5D), and ELISA confirmed higher IGFBP4 concentrations in their follicular fluid (*n* = 12) (Figure 5E). Notably, serum AMH levels were negatively correlated with IGFBP4 abundance (slope = −3.093, R^2^ = 0.65, p = 0.002) (Figure S2G), linking elevated IGFBP4 expression to diminished ovarian reserve.

## 3 Discussion

In this study, we investigated the role of IGFBP4 as a negative regulator of ovarian function and age‐related fertility decline. IGFBP4 suppresses GC proliferation and is associated with increased follicular atresia through antagonizing YAP signaling. Genetic deletion of *Igfbp4* in GCs enhances folliculogenesis, reduces atresia, and improves reproductive performance of female mice. Clinically, IGFBP4 expression is elevated in aged human GCs and in patients with POI, highlighting its potential as both a candidate biomarker and intervention target for age‐related ovarian dysfunction.

### 3.1 IGFBP4 as a Negative Regulator of Female Fertility and Ovarian Aging

A woman's reproductive lifespan is determined by her initial oocyte reserve at birth and the rate of follicle pool decline [36, 37]. However, both endogenous [38] and exogenous factors [39] can accelerate ovarian reserve depletion and reduce oocyte quality, contributing to ovarian aging. In more severe cases, POI—characterized by elevated gonadotropins such as FSH and irregular or absent menstruation before the age 40—can occur [40]. The associated estrogen‐deficient state increases the risk of cardiovascular disease, diabetes, bone loss, and fractures [1], significantly affecting women's reproductive health and overall well‐being.

Accumulating studies have identified several hallmarks of ovarian aging, including telomere shortening, mitochondrial dysfunction, increased oxidative stress, DNA damage, protein homeostasis disruption, aneuploidy, apoptosis, and impaired autophagy [40, 41]. Based on these mechanisms, a number of intervention strategies have been explored, include antioxidants (e.g., vitamins C and E [42], coenzyme Q10 [43]), drugs that modulate oxidative stress (e.g., growth hormone [44], melatonin [41]), and traditional Chinese medicine. While partially effective in certain populations, these treatments are mostly symptomatic and insufficient to delay ovarian aging mechanistically. Therefore, a detailed exploration of molecular regulators of follicular maintenance and loss remains urgently needed.

Among emerging candidates, IGFBPs have drawn attention because of their role in ovarian follicle dynamics. *IGFBP2* expression is markedly reduced in ovine [45] and bovine [46] GCs during follicular growth, but not in primate or human ovaries [47, 48]. *IGFBP5* expression increases dramatically in GCs from ovine and rat atretic follicles, while it slightly decreases in ovine thecal cells [49]. In rodent ovaries, *Igfbp4* has been reported to localize specifically to atretic follicles, with expression levels positively correlated with the severity of follicular degeneration [21]. In human ovaries, *IGFBP4* is highly expressed in the follicular fluid of arrested or atretic androgen‐dominant follicles but is undetectable in estrogen‐dominant, healthy‐growing follicles [22]. Furthermore, increased proteolytic activity targeting IGFBP4 has been observed in preovulatory follicles in humans, sheep, cattle, and horses [50, 51], suggesting that reduction of IGFBP4 activity may be linked to follicle selection and atresia.

In our study, we observed elevated IGFBP4 expression in GCs from aged monkeys, mice, and women. Although several genes ranked above IGFBP4 in Figure 1D, these top hits likely represent broader transcriptional programs in aging GCs, including genes related to membrane‐associated and cell‐state features (CDH19 [52], GPM6B [53], NRXN1 [54]), interferon‐responsed genes (IFI27 [55], ISG15 [56]), an interferon‐related transcription factor (IRF1) [57], a stress‐responsive immediate‐early gene (FOSB) [58], and a metabolic enzyme (ALDH1A1) [59]. We therefore focused on IGFBP4 for mechanistic follow‐up because of its consistent cross‐species upregulation and tractability for in vivo validation. Using the *Igfbp4‐HA* mouse model, we found that *Igfbp4* expression was clearly detected in the developing and antral follicles of young mice and further increased with age. *Igfbp4*‐positive GCs also exhibited increased nuclear abnormalities, suggesting an association with follicular atresia. Importantly, *Amhr2Cre*‐mediated *Igfbp4* conditional knockout (CKO) mice improved reproductive performance in female mice, as shown by increased corpora lutea, higher pup numbers, and better maintenance of fertility with age. Follicle quantification further revealed a larger growing follicle pool, reduced follicular atresia, and enhanced ovulatory output in CKO mice. Consistently, higher AMH and lower FSH levels in CKO mice further supported improved ovarian endocrine function during aging. Together, these findings identify IGFBP4 as a negative regulator of follicle maintenance, thereby impairing reproductive performance and the maintenance of fertility with age.

### 3.2 IGFBP4‐YAP as a Signaling Axis in the Female Reproductive System

IGFBP4 is recognized for its role in inhibiting cell proliferation through its interaction with IGF1. In the context of endometrial cancer, IGFBP4 mediates the inhibition of IGF1, thereby suppressing IGF1‐induced activation of the AKT and ERK pathways, ultimately leading to reduced cell proliferation [60]. In addition, IGFBP4 can exert IGF‐independent effects by inhibiting canonical Wnt signaling, which in turn suppresses steroidogenesis in GCs [61]. In the present study, we evaluated the impact of IGFBP4 treatment on Wnt and IGF signaling pathways. Although no alterations in Wnt signaling were detected (Figure S2H), suppression of IGF1R phosphorylation was observed (Figure S2I,J). To assess whether the function of IGFBP4 depends on IGF1, we performed EdU assays. IGF1 treatment significantly enhanced cell proliferation, whereas IGFBP4 markedly inhibited it. Notably, co‐treatment with IGF1 and IGFBP4 restored proliferation to a level comparable to the control group, indicating that IGFBP4 antagonizes IGF1 activity by limiting its availability for IGF1R activation (Figure S2K). These findings suggest that IGFBP4 inhibits GC proliferation by suppressing IGF1 signaling. Elevated IGFBP4 may bind IGFs and reduce their bioavailability, thereby limiting IGF‐driven GC growth [62]. The signals responsible for IGFBP4 induction in aging/POI GCs remain unclear, but previous studies suggest that genotoxic stress [63], inflammatory signals (e.g., TNF‐α [64]), and growth‐factor (e.g., TGF‐α [65]) may contribute to IGFBP4 upregulation in the ovarian microenvironment.

Previous research has highlighted the pivotal role of the Hippo‐YAP signaling pathway in regulating GC proliferation and follicle development [66]. YAP1 is notably overexpressed in human granulosa tumor cells, where it facilitates proliferation and inhibits differentiation. Additionally, studies have demonstrated that knockout of YAP1 in mouse GCs results in reduced ovarian size, fewer corpora lutea, and pronounced subfertility [18]. These findings support the hypothesis that IGFBP4 inhibits GC proliferation by antagonizing the YAP pathway.

In our study, short‐term IGFBP4 treatment increased the proportion of cells with predominant YAP localization and increased YAP phosphorylation. Conversely, *Igfbp4* knockdown increased YAP nuclear localization and upregulated downstream YAP target genes. This localization‐based regulation was further supported in vivo. GCs isolated from *Amhr2‐Cre; Igfbp4^fl/fl^* ovaries showed reduced YAP phosphorylation and a lower cytoplasmic YAP signal ratio, while total YAP levels remained largely unchanged. The upregulation of YAP target genes in CKO ovaries further indicated enhanced YAP transcriptional output. Together, these findings suggest that IGFBP4 restrains GC proliferation mainly by reducing YAP nuclear localization and downstream YAP transcriptional activation.

### 3.3 IGFBP4 as a Biomarker for the Prediction and Treatment of POI

There is growing interest in identifying biomarkers for POI, a common reproductive endocrine disorder in women of reproductive age that significantly affects physical, mental, and reproductive health. Previous studies have identified UBE2C as a key contributor to POI through analysis of differentially expressed genes and weighted gene co‐expression network analysis (WGCNA) [67]. Additionally, high‐mobility group protein B2 (HMGB2) has been proposed as a novel candidate gene for POI [68].

In the present study, IGFBP4 expression in GCs was significantly elevated in older women undergoing oocyte retrieval compared with younger individuals. IGFBP4 levels were also higher in the follicular fluid of patients with POI than in women without POI. Moreover, follicular fluid IGFBP4 concentrations negatively correlated with AMH, a well‐established indicator of ovarian reserve, suggesting that IGFBP4 reflects impaired granulosa‐cell function and diminished ovarian capacity.

While these findings support the clinical relevance of IGFBP4, its diagnostic performance, including sensitivity, specificity, and clinically meaningful cut‐off values, requires rigorous evaluation in large, multi‐center prospective cohorts. At the current stage, IGFBP4 is best considered a complementary biomarker, and future validation will be essential to define its role in POI assessment and clinical decision‐making.

## 4 Conclusion

In summary, our in vitro and in vivo findings identify IGFBP4 as a key regulator of GC function and a potential contributor to ovarian aging by modulating YAP subcellular localization and downstream YAP target gene expression. By integrating transcriptomic analyses from cynomolgus monkeys, functional validation in *Amhr2‐Cre*‐mediated *Igfbp4* conditional knockout mice, and clinical data from human patient samples, our study provides cross‐species evidence linking IGFBP4 to age‐related ovarian dysfunction. This multi‐model approach enhances the translational value of our findings and supports the potential of IGFBP4 as both a candidate biomarker for conditions such as POI.

Nonetheless, several questions remain. Although follicular atresia is often associated with GC apoptosis, our in vitro data did not reveal a consistent direct pro‐apoptotic effect of IGFBP4. In addition, while *Igfbp4* deletion improved follicular development and endocrine parameters, a direct effect of IGFBP4 on oocyte quality cannot be excluded, and its impact on oocyte competence—including chromosomal integrity and mitochondrial function—still needs to be defined. The broader effects of *Igfbp4* deletion on systemic physiology, including hypothalamic‐pituitary‐ovarian axis stability and metabolic homeostasis, also merit further study. It will also be important to clarify the upstream regulators of IGFBP4, its expression dynamics across the reproductive lifespan, and the feasibility of pharmacological targeting. Collectively, these findings provide a foundation for developing IGFBP4‐based strategies to mitigate age‐related decline in reproductive performance and fertility maintenance.

## 5 Materials and Methods

### 5.1 Experimental Mice

Female mice aged 1–20 months were used in this study. *Igfbp4*‐HA*, Amhr2‐Cre*;*Igfbp4^fl/fl^*, *Igfbp4^fl/fl^*, and *Rosa26^mTmG^* mice were provided by the Chinese Academy of Sciences Center for Excellence in Molecular Cell Science (CEMCS).

For in vitro culture experiments, GCs were sorted from healthy virgin female *Rosa26^mTmG^*, wild‐type ICR, or C57BL/6 mice. For in vivo experiments, female *Amhr2‐Cre*; *Igfbp4^fl/fl^* and *Igfbp4^fl/fl^* mice were used for mating and assessment of reproductive function. *Igfbp4*‐HA mice were used for Igfbp4 immunofluorescent staining.

The PCR program was as follows: initial denaturation at 94°C for 5 min; 35 cycles of denaturation at 94°C for 30 s, annealing at 59°C for 30 s, and extension at 72°C for 1 min; followed by a final extension at 72°C for 10 min Table 1.

**TABLE 1:** Genotyping primer sequences.

Genotyping	Forward primer (5’ to 3’)	reverse primer (5’ to 3’)
Igfbp4‐HA	GATCCTGCCTCTGGAAGCTC	ATCCGGCACATCATACGGAT
Igfbp4‐WT	GCTCTCAATGTCACCTGGCT	CTGGCAGGTCTCACTCTTGG
Amhr2‐Cre	TGCCACGACCAAGTGACAGCAATG	AGAGACGGAAATCCATCGCTCG
*Igfbp4* flox‐5’	CTACTGTTCCTCCTATCTCCTTGAGC	GATTATCCAGCCTGTCACTTCGCCC
*Igfbp4* flox‐3’	GATCCAGAGCCTGATCCACACAC	CATATACCCAAGGGGTCTGCAACTCG
Rosa26^mTmG^	CTCTGCTGCCTCCTGGCTTCT	/
Rosa26^mTmG^‐WT	/	CGAGGCGGATCACAAGCAATA
Rosa26^mTmG^‐mT	/	TCAATGGGCGGGGGTCGTT

All animal procedures were approved by the Animal Care and Use Committee of the Shanghai Institute of Biochemistry and Cell Biology (SIBCB), Chinese Academy of Sciences, under project license number IBCB0065. Mice were housed in the SIBCB animal facility under SPF conditions with a 12‐h light/dark cycle at room temperature, in accordance with institutional guidelines and ethical regulations, and were provided regular chow and water by facility staff.

### 5.2 Patients

All procedures involving patients were approved by the Institutional Ethics Committee of the International Peace Maternity and Child Health Hospital (Shanghai, China) (Approval No. GKLW‐A‐2024‐050‐01). A total of 54 female infertile patients undergoing oocyte retrieval at the same hospital were recruited. Written informed consent was obtained from each participant.

Participants were categorized into three groups: a young control group (<35 years), an older control group (40–50 years), and a POI group. POI diagnosis was based on oligo/amenorrhea lasting at least 4 months and elevated serum FSH levels (>25 IU/L) on two separate occasions more than 4 weeks apart. Age‐specific ovarian reserve criteria were also applied: for women under 35 years, AMH < 1.2 ng/mL and antral follicle count (AFC) < 3–5; for women aged 35–40 years, AMH < 1.0 ng/mL and AFC < 3. A summary of demographic features, age distribution, ovarian reserve indices, and serum hormone levels of all participants is provided (Table S1). Each patient underwent chromosomal analysis, pelvic ultrasound, and thorough medical history evaluation. Patients with chromosomal abnormalities, prior ovarian surgery, chemotherapy, radiotherapy, or autoimmune disorders were excluded. PA (primary amenorrhea) was defined as the absence of menarche before age 16, and SA (secondary amenorrhea) referred to the occurrence of at least one spontaneous menstrual cycle. Patient ages ranged from 22 to 48 years. All patients self‐identified as female, and both ultrasound and karyotype confirmed their sex.

### 5.3 Human Follicular Fluid Collection and Granulosa Cells Isolation

After collecting oocyte‐cumulus complexes from aspirated follicular fluid, the remaining fluid containing GCs was brought to the laboratory for hormone testing and GC isolation. Follicular fluid samples were filtered through a 100 µm cell strainer and centrifuged at 4°C for 5 min. The supernatant was used to determine protein concentration using a Human IGFBP4 ELISA Kit (R&D Systems, USA), following the manufacturer's instructions.

Cell pellets were treated with red blood cell lysis buffer (Sigma) for 5 min, then resuspended in phosphate‐buffered saline (PBS) containing 5% fetal bovine serum (HyClone, USA). Cells were isolated using a Histopaque 1077 density gradient (Sigma–Aldrich, USA). Collected human GCs were cultured in DMEM/F12 medium supplemented with 10% FBS and 100 U/mL penicillin/streptomycin.

### 5.4 Tissue Processing and Follicle Counting

For follicle counting, ovaries were collected at the proestrus stage following euthanasia by CO_2_ asphyxiation, then washed, fixed in 4% PFA overnight, embedded in paraffin, and sectioned continuously at 5 µm thickness. Every fifth section was stained with hematoxylin and eosin. Counting was performed by operators blinded to the treatment or control group.

Follicles were counted in every fifth section to avoid duplication. Primordial follicles were defined as oocytes surrounded by a single layer of flattened or mixed flattened and cuboidal GCs. Primary follicles had an oocyte surrounded by a single layer of cuboidal GCs. Secondary follicles featured a larger oocyte and more than one GC layer with no visible antrum. Early antral follicles exhibited emerging antral spaces, while antral follicles had clearly defined antra. Preovulatory follicles were the largest and contained a cumulus GC layer. Atretic follicles were identified by degenerate or fragmenting oocytes [69].

### 5.5 EdU Labeling

Half of the culture medium in granulosa cell (GC) in vitro cultures was aspirated and replaced with growth medium containing 0.2 mg/mL EdU (Invitrogen). Cells were incubated with EdU for 1 h, followed by fixation in 4% paraformaldehyde for 15 min at room temperature. EdU labeling was performed according to the manufacturer's protocol (Click‐iT EdU Imaging Kit with Alexa Fluor 488, Invitrogen). After fixation, cells were washed three times with PBS (10 min each), and EdU signal development was conducted following the kit instructions. Subsequently, samples were blocked in blocking buffer for 1 h at room temperature, followed by antibody staining and mounting with mounting medium for imaging and quantification.

### 5.6 Immunohistochemistry

For section staining, ovarian tissues were fixed in 4% PFA at room temperature for 15 min, followed by three washes with PBS. Tissues were dehydrated in 30% sucrose at 4°C overnight and embedded in an Optimum Cutting Temperature compound. Sections (16–18 µm) were incubated in 0.1% or 0.5% Triton X‐100 diluted in PBS (PBST) for 20 min and then blocked for 1 h using 10% FBS in PBST.

Sections were incubated with primary antibodies diluted in blocking buffer at 4 °C overnight, followed by three 20‐min washes. After washing, the sections were incubated with secondary antibodies and 4′,6‐diamidino‐2‐phenylindole (DAPI) diluted in a blocking buffer for 2 h at room temperature in the dark. Finally, the sections were washed three times (for 20 min each) and subsequently mounted with a mounting medium.

### 5.7 RNA‐seq and Quantitative Real‐Time PCR Analysis

Total RNA was extracted from granulosa cells (GCs) using Trizol Reagent (Thermo Fisher Scientific), following the manufacturer's instructions. RNA concentration and purity were assessed using a NanoDrop ND‐1000 spectrophotometer (Thermo Fisher). Granulosa cells were isolated from the ovaries of five 2‐month‐old female mice, pooled to minimize inter‐animal variation, and infected with lentivirus encoding one of two independent shRNA constructs targeting Igfbp4. RNA was collected 48 h post‐infection for transcriptome analysis. RNA‐seq libraries were prepared using a standard Illumina TruSeq protocol, and sequencing was performed by Novogene (Beijing, China) on the Illumina NovaSeq 6000 platform with 150 bp paired‐end reads. Differential gene expression analysis was conducted using the DESeq2 R package (v1.20.0). Significantly altered genes (adjusted p‐value < 0.05 and fold change >1.5) were used for downstream pathway enrichment and visualization. RNA‐seq data are publicly available at http://www.biosino.org/node/index, under accession number OEP00005958.

For qPCR, cDNA was synthesized from equal amounts of RNA using the SuperScript III kit. Gene expression was validated using qPCR on a StepOne Plus system (Applied Biosystems) with Power SYBR Green PCR Master Mix. Expression levels were normalized to *Gapdh*. The PCR cycling conditions were: 10 min at 95°C for initial denaturation, followed by 40 cycles of 15 s at 95°C (denaturation) and 1 min at 60°C (annealing and extension), followed by a melt curve analysis. The primers used are listed in Table 2:

**TABLE 2:** Primer sequences used for qantitative real‐time PCR (qPCR) in the study.

Gene	Forward primer (5’ to 3’)	reverse primer (5’ to 3’)
Ms‐Cyr61	GCTCAGTCAGAAGGCAGACC	GTTCTTGGGGACACAGAGGA
Ms‐Yap1	GCCATGCTTTCGCAACTGAA	CAAAACGAGGGTCCAGCCTT
Ms‐Birc5	AGAACAAAATTGCAAAGGAGACCA	GGCATGTCACTCAGGTCCAA
Ms‐Ctgf	CTGCCTACCGACTGGAAGAC	CATTGGTAACTCGGGTGGAG
Ms‐Ankrd1	GGATGTGCCGAGGTTTCTGAA	GTCCGTTTATACTCATCGCAGAC
Ms‐IGFBP4	AGAAGCCCCTGCGTACATTG	TGTCCCCACGATCTTCATCTT
Ms‐Gapdh	TGTGATGGGTGTGAACCACGAGAA	CTGTGGTCATGAGCCCTTCCACAA
HU‐IGFBP4	GGTGACCACCCCAACAACAG	GAATTTTGGCGAAGTGCTTCTG
HU‐GAPDH	AACAGCGACACCCACTCCTC	CATACCAGGAAATGAGCTTGACAA

### 5.8 Single‐Cell Transcriptomic Data From Macaque and Homo Sapiens

Single‐cell RNA‐Seq data from cynomolgus monkey (Macaca fascicularis) ovaries were downloaded from GEO (GSE130664) [19]. Raw count matrices from young and aged monkeys were imported into Seurat (v5.0) in R and processed with a standard pipeline (quality control, normalization, highly variable gene selection, scaling, PCA, clustering and UMAP embedding). Datasets from young and aged animals were integrated using Seurat integration functions to obtain a joint atlas. Granulosa cell clusters were identified based on canonical markers and subsetted. Differential expression between granulosa cells from young and aged monkeys was computed with FindMarkers (Wilcoxon rank‐sum test, adjusted P values), and volcano plots were generated in R using ggplot2.

Publicly available human ovarian single‐cell RNA‐Seq data were obtained from GEO (GSE255690) [34]. Only samples from the young and old groups (3 young vs 3 old donors) were included in this study. Raw count matrices were imported into Seurat (v4.x) in R and processed with a standard workflow. Samples from young and old donors were then integrated using Seurat's integration workflow to construct a combined reference. Granulosa cells were identified using established marker genes and subsetted for analysis. Differential expression between young and old groups was computed with FindMarkers (Wilcoxon rank‐sum test with multiple‐testing correction), and volcano plots were generated in R using ggplot2.

### 5.9 Primary Mouse Granulosa Cell and KGN Cell Line Culture Protocols

Mouse GCs were isolated as previously described [20]. Briefly, ovaries from *Rosa26^mTmG^* mice were harvested, washed in cold PBS, and separated from the ovarian bursa by microdissection under a stereomicroscope. Single cells were obtained using established protocols [70]. Minced ovarian tissue was digested with digestion buffer consisting of RPMI 1640 (Thermo Fisher, Cat#12633‐012), 5% fetal bovine serum (FBS; HyClone, Cat#SH30071.03), 1% penicillin‐streptomycin (Thermo Fisher, Cat#15140122), 25 mM HEPES, and 300U/mL collagenase IV (Worthington, Cat#LS004189), incubated at 37 °C with shaking at 100 rpm for 1 h.

The cell suspension was treated with red blood cell lysis buffer (Sigma, Cat#R7757), then incubated with 0.05% Trypsin‐EDTA (Thermo Fisher, Cat#25200056) and DNase I (Sigma, Cat#D4263). The resulting single‐cell suspension was filtered through a 70 µm cell strainer.

Cell sorting was performed using FACSJazz (BD Biosciences). Upon analysis of dissociated *Rosa26^mTmG^* ovarian cells on a FACS contour plot (with Tomato fluorescence on the X‐axis), two populations were identified. Based on fluorescence intensity, cells were gated into “mTom‐low” (dimmer) and “mTom‐high” groups. Cells in the mTom‐low population were collected as a granulosa cell‐enriched fraction. Post‐sort purity was routinely assessed and confirmed to be >95%. For cell culture, sorted GCs were resuspended in DMEM/F12 medium (Thermo Fisher, Cat#11320033) supplemented with 10% FBS (HyClone, Cat#SH30071.03) and 1% penicillin‐streptomycin (Thermo Fisher, Cat#15140122).

Human granulosa‐like tumor cell line KGN (RRID: CVCL_0375) was obtained from Fenghui Biotechnology (Hunan, China, Cat# CL0544) and authenticated by short tandem repeat (STR) profiling. KGN cells were cultured in DMEM/F12 medium (Thermo Fisher, Cat# 11320033) supplemented with 10% FBS (HyClone, Cat#SH30071.03) and 1% penicillin‐streptomycin (Thermo Fisher, Cat#15140122) and incubated at 37°C in a 5% CO_2_.The cell line was obtained from a certified supplier and maintained under sterile conditions throughout the study. No contamination was observed during routine culture. The KGN line is widely used to model granulosa cell function in vitro, and its use does not affect the validity of the results.

### 5.10 Lentiviral Production and GC Infection

The shRNA sequences targeting *Igfbp4* were cloned, and lentiviruses were produced in 293T cells via co‐transfection of transfer vectors using Opti‐MEM (Thermo Fisher, Cat#31985070). Transfection media were replaced 12 h post‐transfection. Viral supernatants were collected at 48 h and filtered through a 0.45 µm membrane. For GC infection, the packaged virus was diluted in GC culture medium along with 1:100 polybrene (Sigma, Cat#TE1003) and applied for 12 h. The infected GCs were harvested 3 days post‐infection.

### 5.11 Fertility Testing

Female mice (Ctrl and CKO; *n* = 7 females per genotype) were continuously mated with proven fertile males (8‐10 weeks old) in a 2:1 female‐to‐male ratio (two females and one male per cage). Cages were checked weekly, and the number of newly delivered pups since the previous check was recorded. To assign offspring to maternal genotype in co‐housing cages, all pups were genotyped for *Amhr2‐Cre*, and newly delivered pups were counted separately for Ctrl vs CKO females. Weekly counts were cumulated for each female to generate the cumulative number of pups delivered per female over time (reproductive curve). Mating was continued until females were considered reproductively inactive, defined as no newly delivered pups for 8 consecutive weekly checks (2 months), or until the predefined (13 months of age).

### 5.12 Measurements of Serum Hormones

To minimize variability due to the estrous cycle, blood was collected from the orbital venous sinus at the diestrus stage under anesthesia with Avertin (8 mL/kg body weight, intraperitoneally) from young (3–6 months; n = 6 per group) and old (12–14 months; n = 7 per group) CKO and Ctrl mice. Mouse AMH (WestTang, F10054) and FSH (WestTang, F10450) levels were measured using respective ELISA kits according to the manufacturer's protocols.

### 5.13 Quantification and Statistical Analysis

All quantitative data are presented as mean ± standard deviation (SD), unless otherwise specified. Each experiment was independently repeated at least three times, with pooled data shown in the figures. Sample size (n) for each experiment is indicated in the corresponding figure legend. Statistical analyses were performed using GraphPad Prism 9 (GraphPad Software, USA). For comparisons between two groups, a two‐tailed unpaired Student's t‐test was used. For multiple group comparisons, one‐way ANOVA followed by Tukey's post‐hoc test was applied. A significance level of α = 0.05 was used for all tests. All assumptions for parametric tests, including normality and homogeneity of variance, were assessed prior to analysis. P values are reported in the figure legends, with significance denoted as follows: n.s. p > 0.05; ^∗^ *p* < 0.05; ^∗∗^ *p* < 0.01; ^∗∗∗^ *p* < 0.001.

## Conflicts of Interest

The authors declare no conflicts of interest.

## Supporting information




**Supporting File**: advs76580‐sup‐0001‐SuppMat.docx.

## Data Availability

Mass spectrometry data supporting this study are publicly available at http://www.biosino.org/node/index, under accession number OEP00005958. All other data and materials generated in this study are available from the corresponding author upon request.
