# Supplementary material for: Inhibition of IGFBP4 in Granulosa Cells Improves Reproductive Performance and Maintains Fertility With Age via YAP Signaling
Source: Adv Sci (Weinh). 2026 Jul 16:e10226. Online ahead of print. doi: 10.1002/advs.202510226 (PMC13373898; doi:10.1002/advs.202510226)
Supplement: Supplementary file 1 — Supporting File: advs76580‐sup‐0001‐SuppMat.docx. [file ADVS-9999-e10226-s001.docx]

**SUPPLEMENTAL MATERIALS**

Inhibition of IGFBP4 in Granulosa Cells Improves Reproductive Performance and Maintains Fertility with Age via YAP Signaling

Qianhui Hu ^1, 2, #^, Ajun Geng ^3, #^, Ziyuan Li ^4,5, #^, Fanghao Guo ^1, 2, #^, Jingqiang Wang ^3^, Xinyi Chen ^6^, Meiling Zhang ^1, 2^, Yi Arial Zeng ^3,6 *^ and Wen Li ^1, 2 *^

^1^ Center for Reproductive Medicine & Fertility Preservation Program, International Peace Maternity and Child Health Hospital, School of Medicine, Shanghai Jiao Tong University, Shanghai, 200030, China.

^2^ Shanghai Key Laboratory of Embryo Original Disease, Shanghai, 200030, China.

^3^ State Key Laboratory of Cell Biology, CAS Center for Excellence in Molecular Cell Science, Institute of Biochemistry and Cell Biology, Chinese Academy of Sciences, University of Chinese Academy of Sciences, Shanghai 200031, China.

^4^ The Center of Reproductive Medicine, Shanghai Changzheng Hospital, Naval Medical University, Shanghai, 200003, China.

^5^ The Center of Reproductive Medicine, Shanghai Changzheng Hospital, Naval Medical University, Shanghai, 200003, China.Hospital of Zhejiang People's Armed Police, Hangzhou, 310051, China.

^6^ Key Laboratory of Systems Health Science of Zhejiang Province, School of Life Science, Hangzhou Institute for Advanced Study, Hangzhou 310024; University of Chinese Academy of Sciences, China.

^#^ These authors contributed equally to this work.

* **Authors for correspondence:**

Wen Li, MD, PhD, Center for Reproductive Medicine & Fertility Preservation Program, International Peace Maternity and Child Health Hospital, School of Medicine, Shanghai Jiao Tong University, Shanghai, 200030, China. Email: liwen@shsmu.edu.cn.

Yi Arial Zeng, PhD, State Key Laboratory of Cell Biology, CAS Center for Excellence in Molecular Cell Science, Institute of Biochemistry and Cell Biology, Chinese Academy of Sciences, University of Chinese Academy of Sciences, Shanghai 200031, China.

Key Laboratory of Systems Health Science of Zhejiang Province, School of Life Science, Hangzhou Institute for Advanced Study, Hangzhou 310024; University of Chinese Academy of Sciences, China. Email: [yzeng@sibcb.ac.cn](mailto:yzeng@sibcb.ac.cn)


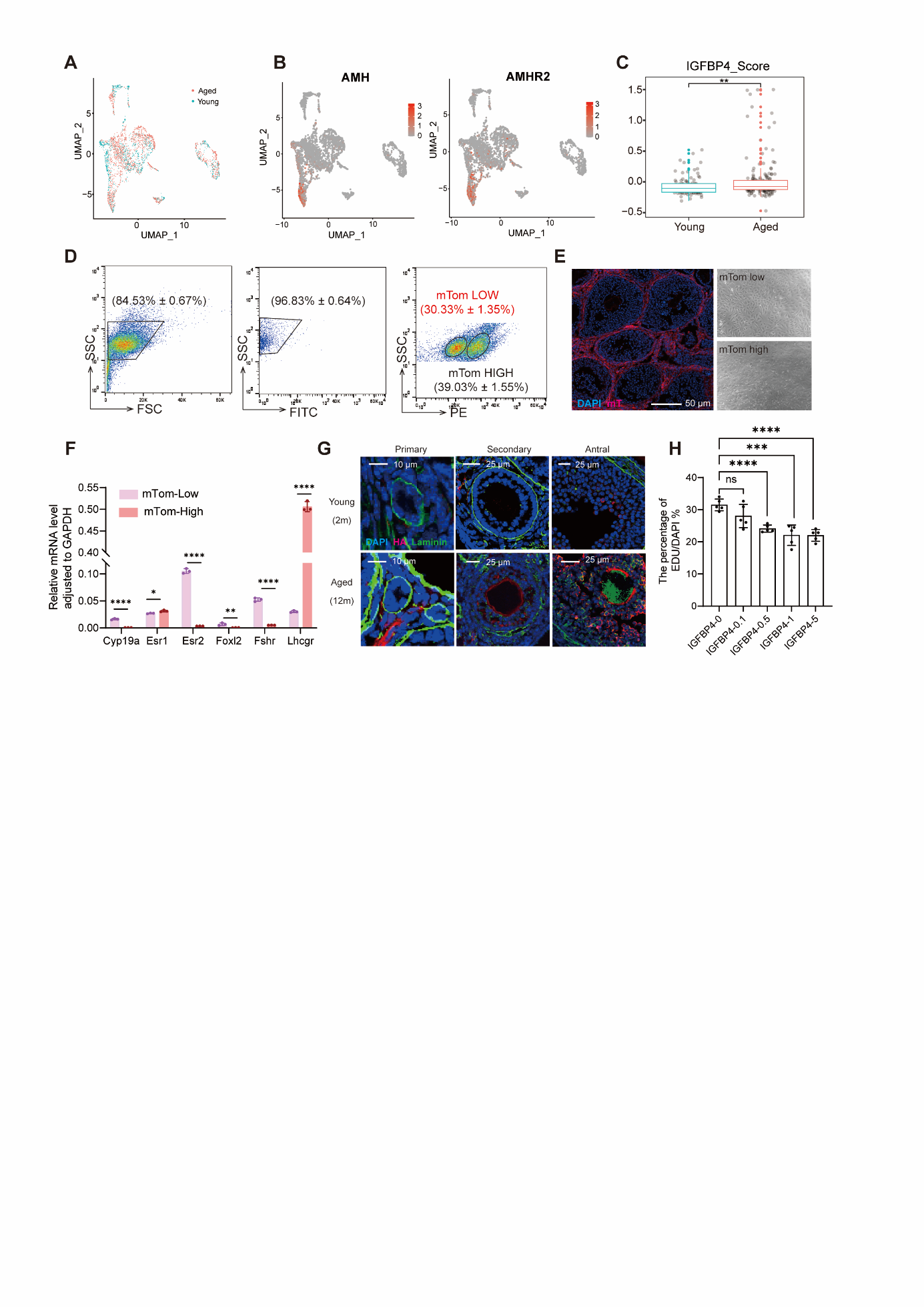


**Figure S1**

**(A)**  UMAP plot of ovarian single-cell transcriptomes color-coded by age group. **(B)** Feature plots showing AMHR2 and AMH expression in cynomolgus monkey GCs. **(C)** Box plot showing the IGFBP4 score of the young and aged groups based on the AddModuleScore function. **(D)** FACS sorting strategy for ovarian cells from *Rosa26^mTmG^* mice based on mTomato fluorescence intensity. GCs were enriched in the mTom-low population. **(E)** Immunofluorescence staining of ovaries from *Rosa26^mTmG^* mice to visualize mTomato signal in ovarian cells. mTom-low and mTom-high cell populations were sorted by FACS and cultured separately for morphological assessment. **(F)** qPCR analysis of *Cyp19a1*, *Foxl2*, *Esr1*, *Esr2*, and *Fshr* in isolated mTom-low and mTom-high ovarian cells verifying enrichment of GC markers in the mTom-low population (n = 3). Mean ± SD. *p < 0.05, **p < 0.01, ****p < 0.0001. **(G)** Higher-magnification views of the corresponding follicles shown in Figure 1G, illustrating IGFBP4-HA expression (red), laminin (green), and DAPI (blue) in primary, secondary, and antral follicles from young (2-month-old) and aged (12-month-old) mice. **(H)** EdU incorporation assay to evaluate the effect of increasing concentrations of recombinant IGFBP4 (0.1–5 μg/mL) on KGN cell proliferation (n=5). Cells were treated with IGFBP4 for 24 h, followed by a 1 h EdU pulse prior to fixation and staining. Mean ± SD. ns, not significant, ***p < 0.001, ****p < 0.0001.

**
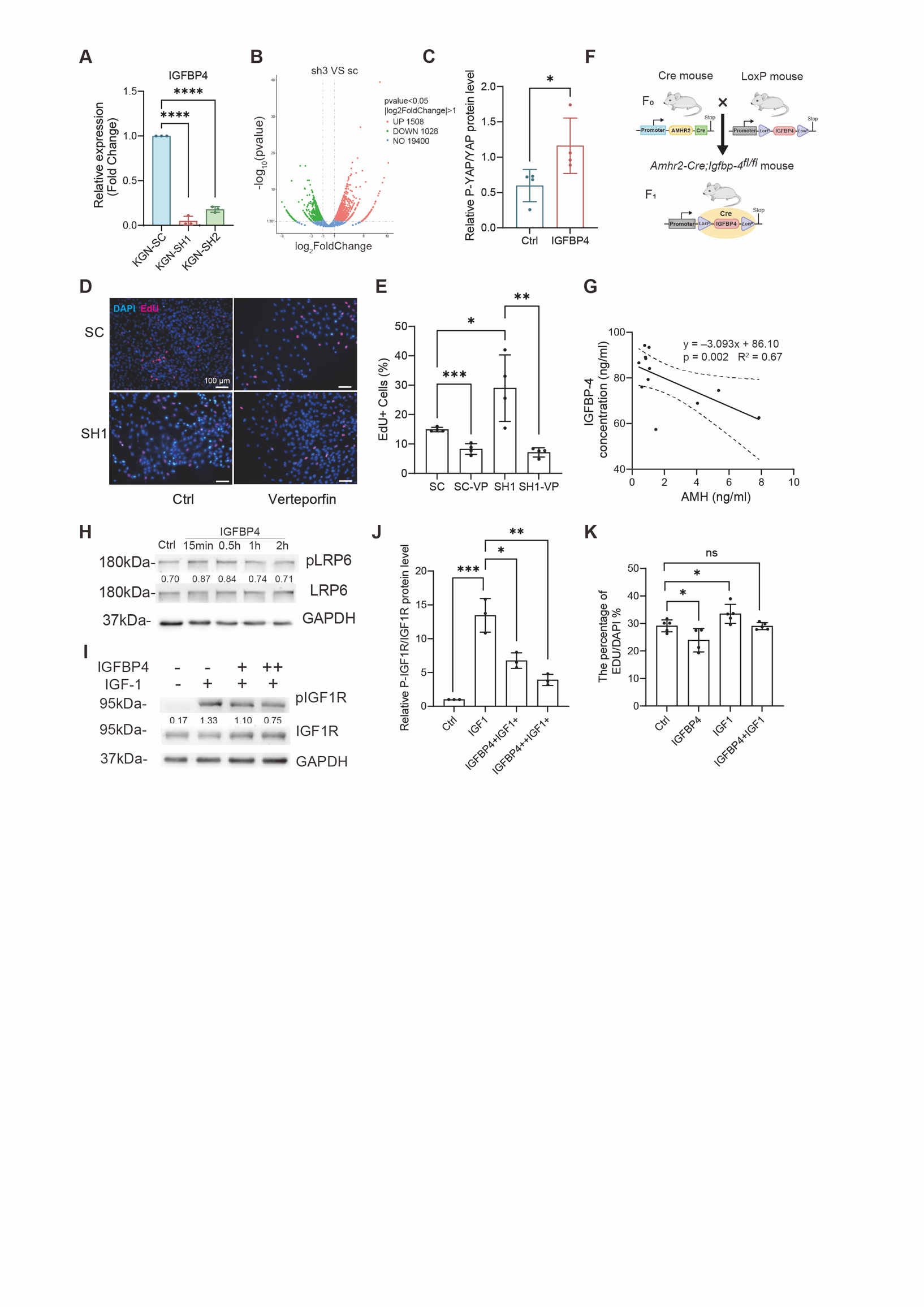
**

**Figure S2**

**(A)** qPCR analysis validated the knockdown efficiency of IGFBP4 in KGN cell line (SH1, SH2). Mean ± SD. ***p < 0.001. **(B)** Volcano plot showing all up- and down-regulated genes. (p < 0.05. |log2FoldChange|>1). **(C)** Densitometric quantification of the p-YAP/YAP ratio in KGN cells after 30 minutes of stimulation with 5 μg/mL IGFBP4 protein. Mean ± SD. *p < 0.05. **(D)** Representative EdU immunofluorescence images of KGN cells transfected with control shRNA (SC) or IGFBP4 shRNA (SH1) and treated with or without Verteporfin (VP). Scale bar, 100 μm. **(E)** Quantification of the percentage of EdU⁺ cells in the indicated groups. Mean ± SD. *p < 0.05, **p < 0.01, ***p < 0.001. **(F)** Schematic diagram showing the strategy to generate an *Amhr2-Cre; Igfbp4^fl/fl^* mouse model. **(G)** Linear regression analysis between AMH and IGFBP4. The best-fit line (y = –3.093x + 86.10) is shown with a 95% confidence interval. The slope was –3.093 (p = 0.002), with X-intercept = 27.84 and 1/slope = –0.323. **(H)** Phosphorylation levels of the Wnt protein LRP6 at different time points after IGFBP4 treatment. The concentration of IGFBP4 was 2 μg/ml. **(I)** Phosphorylation levels of IGF1R after 1 h co-stimulation with IGF1 (100 ng/ml) and IGFBP4 at varying concentrations (+: 2 μg/ml, ++: 10 μg/ml). **(J)** Densitometric quantification of the p-IGF1R/IGF1R ratio after 1 h co-stimulation with IGF1 (100 ng/ml) and IGFBP4 at varying concentrations (+: 5 μg/ml, ++: 10 μg/ml). Mean ± SD. *p < 0.05, **p < 0.01, ***p < 0.001. (K) Quantification of KGN cell proliferation by EdU under different treatments (n=5), KGN cell was treatment with IGF1 (100 ng/ml) and IGFBP4 (5 μg/ml). Mean ± SD. ns, not significant, *p < 0.05.

**Supplementary Table 1. Summary of demographic and clinical characteristics of human participants.**

| Variable | Young group (mean±SD) | Aging group (mean±SD) | POI group (mean±SD) |
| --- | --- | --- | --- |
| n | 25 | 12 | 17 |
| Age (years) | 28.91±2.93 | 44.31 ±2.39 | 32.29±2.64 |
| AMH (ng/mL) | 5.06±2.85 | 1.41± 1.63 | 0.69 ±0.28 |
| Level of FSH (IU/L) | 13.77±4.89 | 20.88±10.77 | 21.16±8.46 |
